# Copper toxicity in a New Zealand dairy herd

**DOI:** 10.1186/2046-0481-67-20

**Published:** 2014-09-23

**Authors:** Howard Johnston, Laura Beasley, Neil MacPherson

**Affiliations:** 1 Ministry for Primary Industries, PO Box 966 Waikato Mail Centre, Hamilton, New Zealand; 2 The Vet Club and Animal Hospital, Marguerita Street Rotorua, New Zealand; 3 Vet Focus, 565 Mahoe Street, Te Awamutu, New Zealand

**Keywords:** Copper, Toxicity, Dairy, Bovine, Sudden death, New Zealand, Herd health, Jersey

## Abstract

Chronic copper toxicity was diagnosed in a Jersey herd in the Waikato region of New Zealand following an investigation into the deaths of six cattle from a herd of 250 dry cows. Clinical signs and post-mortem examination results were consistent with a hepatopathy, and high concentrations of copper in liver and blood samples of clinically affected animals confirmed copper toxicity. Liver copper concentrations and serum gamma-glutamyl transferase activities were both raised in a group of healthy animals sampled at random from the affected herd, indicating an ongoing risk to the remaining cattle; these animals all had serum copper concentrations within normal limits. Serum samples and liver biopsies were also collected and assayed for copper from animals within two other dairy herds on the same farm; combined results from all three herds showed poor correlation between serum and liver copper concentrations.

To reduce liver copper concentrations the affected herd was drenched with 0.5 g ammonium molybdate and 1 g sodium sulphate per cow for five days, and the herd was given no supplementary feed or mineral supplements. Liver biopsies were repeated 44 days after the initial biopsies (approximately 1 month after the end of the drenching program); these showed a significant 37.3% decrease in liver copper concentrations (P <0.02). Also there were no further deaths after the start of the drenching program. Since there was no control group it is impossible to quantify the effect of the drenching program in this case, and dietary changes were also made that would have depleted liver copper stores.

Historical analysis of the diet was difficult due to poor record keeping, but multiple sources of copper contributed to a long term copper over supplementation of the herd; the biggest source of copper was a mineral supplement. The farmer perceived this herd to have problems with copper deficiency prior to the diagnosis of copper toxicity, so this case demonstrates the importance of monitoring herd copper status regularly. Also the poor correlation between liver and serum copper concentrations in the three herds sampled demonstrates the importance of using liver copper concentration to assess herd copper status.

## Background

There are two forms of copper poisoning – acute and chronic. Acute copper poisoning can result from the accidental administration of large quantities of copper (often via oral copper salts, parenteral copper administration, or grazing pasture recently fertilised with copper) [1]. Chronic copper poisoning is associated with the slow accumulation in the liver of smaller amounts of copper ingested over a long period of time, but with no change in blood copper levels. When the liver’s capacity to accumulate copper is overloaded, usually after a stressful event, there is a release of copper into the bloodstream that leads to intravascular haemolysis. Combined with liver damage this causes acute toxicosis and recumbency with affected animals often dying within 24 – 48 hours; these animals show symptoms of profound depression, thirst, anorexia, pale or icteric mucous membranes and haemoglobinuria [1].

Plasma or serum copper concentrations are commonly used as a measure of copper status, but in healthy animals these are poorly correlated with liver copper concentrations, except when liver copper concentrations are extremely low [2]. Sources of copper include pasture, fertiliser, palm kernel extract (PKE), and mineral supplement (oral and injectable). A quarterly review of diagnostic cases received by New Zealand Veterinary Pathology for April to June 2012 reported several cases of copper toxicity in dairy cows; the common factors in all of the cases were PKE feeding, drying off the cows, and restricting feed intake [3].

Copper absorption is affected by several antagonists commonly present in the soil (most notably iron, molybdenum and sulphur) but there is also evidence that high levels of dietary zinc can inhibit copper absorption [4]. Cattle in New Zealand are supplemented with high levels of oral zinc throughout summer and autumn to prevent facial eczema. Facial eczema (FE) is a disease of grazing ruminants caused by ingestion of spores of the fungus Pithomyces chartarum, which produce the hepatotoxic mycotoxin sporidesmin [5]. The presence of FE and the high levels of zinc supplementation required to prevent it add another factor to consider when assessing copper supplementation in New Zealand herds which is not present in Ireland.

The aim of this paper is to present a case of sudden death due to copper toxicity seen in a dairy herd in the Waikato region of New Zealand, and to discuss appropriate diagnosis, management, and investigation of this condition.

## Case presentation

### History

In early May 2012 a herd of approximately 250 Jersey cows were dried off, accompanied by a diet change (grass intake was restricted, maize silage feeding was increased and feeding of PKE was stopped). The herd owner had historically been maintaining high levels of copper supplementation as he perceived the farm to have problems with copper deficiency. However, liver samples taken from groups of five cull cows at slaughter in 2002, 2005 and 2010 showed group average liver copper (Cu(L)) concentrations within the reference range (95 – 2000 μmol/kg wet weight [6]).

This farm operated a seasonal calving system so individual dry period length varied depending on calving date, but the minimum dry period for this herd was 80 days. This herd of cows (Dairy One herd) was part of a larger dairy farm consisting of three dairy herds managed separately. Four days following dry off two cows (cows 1 & 2) were found dead and a third cow (cow 3) was noticed to be weak; this sick cow died within 24 hours despite veterinary treatment. Over the next four days a further three cows (cows 4 to 6) were observed to be sick and subsequently died, bringing the total of dead cows to six.

### Investigation

**Figure 1:**
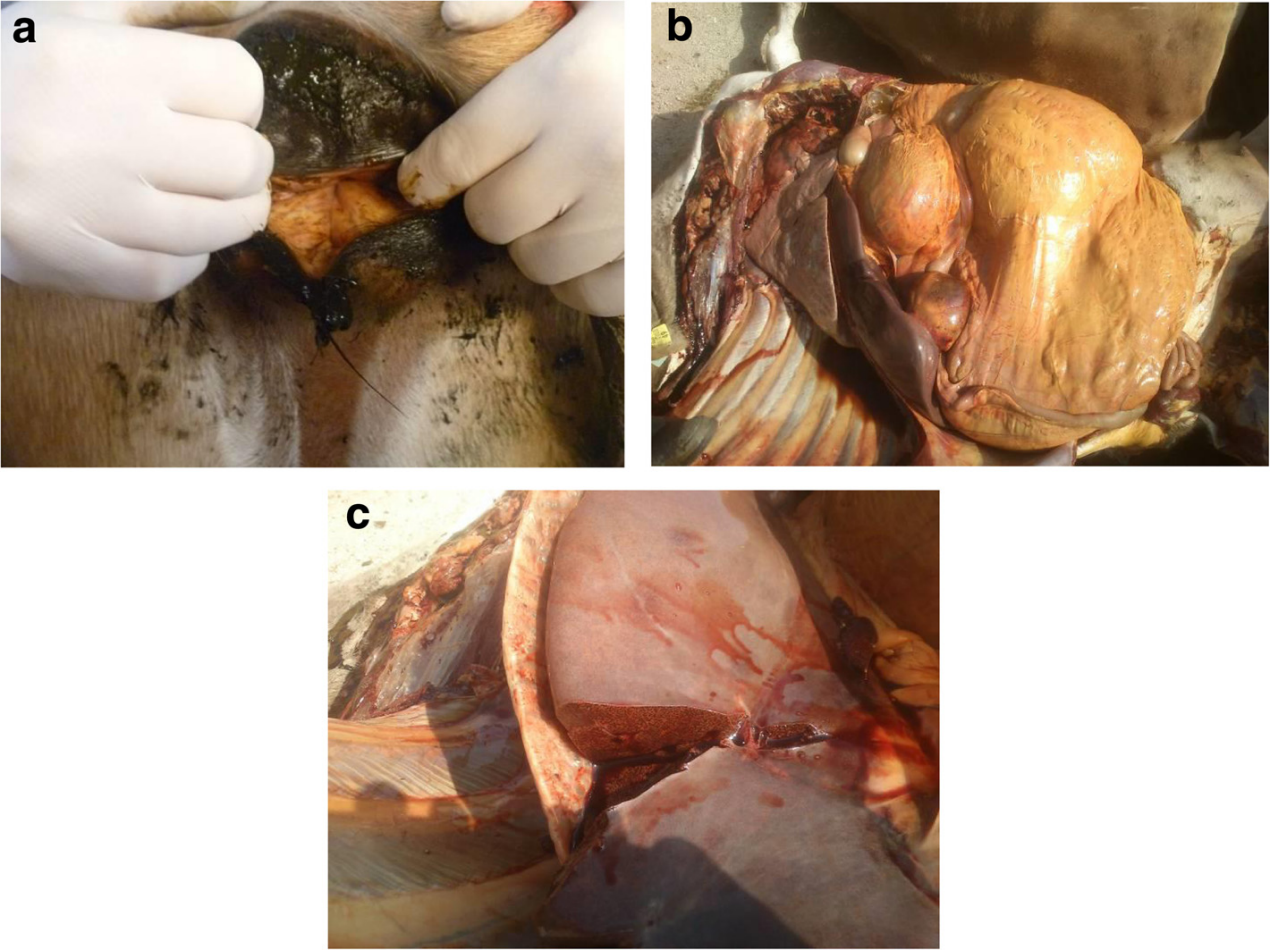
**Gross lesions seen on post-mortem examination of cow 3. (a)** Icteric vaginal mucous membranes. **(b)** Yellow pigmented tissues throughout carcase. **(c)** Rounded bulging liver margins.

Examination of both dead cows (cows 1 & 2) and the sick cow (cow 3) revealed icteric mucosae on all three animals. On the first visit ocular fluid was sampled from each of the dead cows to rule out metabolic diseases and nitrate toxicity, and a blood sample was taken from cow 3 for haematology and biochemistry. Initial laboratory results from the blood and ocular fluid ruled out hypocalcaemia, hypomagnesaemia and nitrate toxicity but on serum biochemistry both gamma-gluatmyl transferase [7] (GGT) and glutamate dehydrogenase [7] (GLDH) activities were markedly increased, suggesting a hepatopathy. Follow up testing revealed a raised serum copper [8] (Cu(S)) concentration (67 μmol/l; reference 8-20 μmol/l [6]) on the blood sample taken from cow 3.Cow 3 died within 24 hours of displaying clinical signs and a post-mortem examination was carried out; gross findings were bulging liver margins and a zonal (nutmeg) appearance to the liver. The whole carcase had an icteric appearance (see Figure 1), although this was difficult to interpret due to the presence of carotenoid pigmentation commonly seen in Jersey cattle. Findings of histological examination were marked acute periacinar necrosis in the liver and evidence of a haemoglobinuric nephrosis.

Once a hepatopathy was suspected the entire herd was examined and mucous membrane colour checked by farm staff; approximately ten cows with icteric mucous membranes were segregated. Mucous membrane colour was re-examined later the same day by a veterinarian and had apparently returned to normal in all but one of these cows (cow 4); this cow subsequently became clinically affected and died along with two other cows (cows 5 and 6). Ante-mortem blood samples and post-mortem kidney and liver samples were taken from each of the remaining three cows that died (cows 4-6) and assayed for serum GGT, serum GLDH, and copper concentration (liver [8], kidney [7] and serum). These results supported a diagnosis of copper toxicity (see Table 1).

**Table 1:** Concentration of Cu in serum, liver and kidney, and the activities of serum GGT and GLDH in the last three cows (cows 4 to 6) to die in this copper toxicity outbreak

	**Cow 4**	**Cow 5**	**Cow 6**	**Normal range**
**Kidney Cu**	120	820	310	<157 μmol/kg [9]
**Liver Cu**	2300	2400	2700	95 – 2000 μmol/kg wet tissue [6]
**GGT**	201	192	299	<40 IU/l^a^
**GLDH**	240	1258	236	<50 IU/l^a^
**Serum Cu**	34.0	99.0	52.0	8 – 20 μmol/l [6]

^a^– source is unpublished data from Ruakura Animal Health Laboratory.

### Herd status

Following the conclusion that these deaths were related to copper toxicity the decision was made to assess the copper status of the affected herd by sampling ten healthy animals chosen at random. Core liver biopsies were collected under local anaesthesia [10] and analysed to assess the level of copper stored by the cows, while blood samples were assayed for copper concentration, as well as GGT and GLDH for liver damage (subclinical liver damage preceeds the acute haemolytic crisis).

Interestingly, the average Cu(L) concentration for the last three animals (cows 4-6) that died of copper toxicity was slightly lower (2467 μmol/kg fresh tissue) than that for the healthy group (2620 μmol/kg fresh tissue), possibly reflecting release of copper into the bloodstream. Correspondingly the average Cu(S) concentration for these clinically sick animals was markedly elevated (61.2 μmol/l) compared to the healthy group (12.9 μmol/l). GGT and GLDH levels were elevated for the healthy group, showing evidence of liver damage (see Table 2). High Cu(L) concentrations were not well correlated with raised GGT activities (Spearman’s Rank Correlation Coefficient 0.25).

**Table 2:** Serum and liver copper concentrations, and serum GGT and GLDH activities from healthy animals sampled at random from the affected herd

**Cow**	**Serum Cu (μmol/l)**	**Liver Cu (μmol/kg wet tissue)**	**Serum GGT (IU/L)**	**Serum GLDH (IU/L)**
**A**	10.0	1900	31	38
**B**	16.0	**3000**	**225**	**325**
**C**	16.0	**3200**	**118**	**286**
**D**	13.0	**3500**	**167**	**420**
**E**	16.0	Insufficient sample	**236**	**317**
**F**	11.0	**2600**	15	14
**G**	14.0	**3300**	13	32
**H**	12.0	2000	**60**	41
**I**	11.0	**2200**	**115**	**139**
**J**	12.0	2000	**48**	**159**
**K**	11.0	**2500**	46	44
**Mean (standard error of mean)**	12.9 (± 0.7)	**2620 (± 188)**	**98 (± 24)**	**165 (± 44)**
**Reference Range**	8.0 – 20.0 [6]	95 – 2000 [6]	<40 IU/l^a^	<50 IU/l^a^

Values outside the reference range are highlighted in bold.

^a^– source is unpublished data from Ruakura Animal Health Laboratory.

**Figure 2:**
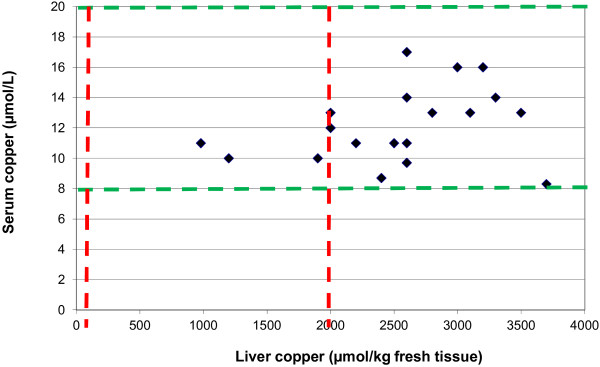
**Liver vs. serum copper concentrations for healthy animals (19 animals sampled from all three herds).** Red lines show normal range for liver copper concentrations, green lines show normal area for serum copper concentrations.

In addition liver biopsies and blood samples to assess herd copper status were taken from the other two dairy herds on the farm (seven cows at Dairy Two and six cows at Dairy Three). The average Cu(L) concentration was raised for the cows at Dairy Two (2800 μmol/kg fresh tissue) and within normal limits (95 – 2000 μmol/kg fresh tissue [6]) for the cows at Dairy Three (1996 μmol/kg fresh tissue). All of these animals had Cu(S) concentrations within the reference range. Despite the majority of the healthy animals sampled from the three herds having raised Cu(L) concentrations, all of them had Cu(S) concentrations within the reference range (see Table 2) and the correlation between Cu(L) and Cu(S) concentrations was poor (Spearman’s Rank Correlation Coefficient 0.40) (see Figure 2). Accumulation of copper in the liver can lead to a sudden release of copper into the blood stream and cause symptoms of copper toxicity due to haemolysis and liver damage [1].

### Risk management

Raised Cu(L) concentrations and evidence of liver damage in healthy animals indicated a risk to the remainder of the herd so the decision was taken to start a daily drenching program with ammonium molybdate and sodium sulphate. The aim was to reduce the amount of copper being stored in the liver and prevent further deaths from copper toxicity. Feeding of PKE had already stopped and the herd was no longer receiving any mineral supplementation.

Dose rates of 0.5 g/cow/day of ammonium molybdate and 1 g/cow/day sodium sulphate were used, as this has been shown to deplete liver copper reserves [11]. Practical considerations meant that drenching was the best method to administer molybdenum/sulphur supplementation, and the herd was only drenched for five days. Liver biopsies and blood samples were repeated on the “healthy group” previously sampled from the affected herd approximately 1 month after the end of the drenching program to try and assess any changes in herd copper status.

Following the drenching program there were no further deaths and individual Cu(L) concentrations fell in the cows sampled, bringing the five out of the eight of animals sampled post-drenching back within the reference range. Taking into account only cows with both pre- and post-drenching Cu(L) concentrations available, the average reduction in liver copper concentration was 37.3% (see Table 3). This difference was statistically significant using a Wilcoxin Signed Rank test (P < 0.02), indicating that the management strategies had been effective at reducing liver copper stores. As Cu(L) concentrations were also raised at Dairy Two this herd was supplemented with ammonium molybdate and sodium sulphate via the feed, but no follow-up liver biopsies were obtained to determine the effect on liver copper reserves.

**Table 3:** Mean changes in Cu(l) concentration of cows over 44 days following the removal of all Cu supplements and drenching with 0.5 g ammonium molybdate and 1.0 g sodium sulphate for five days

**Cow**	**Liver Cu pre drenching (μmol/kg wet tissue)**	**Liver Cu post drenching (μmol/kg wet tissue)**	**% reduction in liver Cu**
**B**	3000	1500	50.0%
**C**	3200	2100	34.4%
**D**	3500	1600	54.3%
**F**	2600	1700	34.6%
**G**	3300	2600	21.2%
**H**	2000	1100	45.0%
**J**	2000	1700	15.0%
**Mean (standard error of mean)**	2800 (± 232)	1757 (± 180)	37.3%

Only cows with both pre- and post-drenching liver biopsy results available are included.

### Sources of copper

Once a diagnosis of copper poisoning was reached an investigation was undertaken into possible sources of copper, but due to poor recording it was difficult to obtain an accurate diet and supplement history for the herd. However an attempt was made to estimate dietary copper intake for the herd. Three of the four pastures sampled had medium levels of copper (9-11 mg/kg dry matter (DM); medium range 10-12 mg/kg DM) while one pasture had a higher copper level (17 mg/kg DM); this paddock was grazed by the herd immediately prior to the copper poisoning outbreak and was an effluent paddock. Effluent paddocks are used to spread cow faecal material and urine collected from handling areas (e.g. collecting yard); these paddocks are not grazed for a minimum of 10 days after effluent application. A footbath containing copper had been used at the exit to the milking shed during the season, so it is possible that this contributed to the high copper levels measured in the effluent paddock.

The herd had also been fed on maize silage and PKE; the PKE had a copper level of 22 mg/kg DM but no maize silage sample was available for analysis. Also the herd had been supplemented with a customised mineral blend containing 370 mg elemental copper/cow/day (120 mg as copper glycine and 250 mg as copper sulphate) when used at recommended doses (5 g/cow/day). Due to poor recording it was not possible to determine the exact dose of mineral blend used or over what period it was used. A lactating Jersey cow producing 20 litres of milk/day under reasonable New Zealand pasture conditions (normal iron, sulphur and molybdenum) has a daily copper requirement of approximately 136 mg [6]. For this herd:

•Pasture = 13 kg DM @ 10 mg/kg DM = 130 mg Cu

•PKE = 3 kg DM @ 22 mg/kg DM = 66 mg Cu

•Mineral blend @ 5 g/cow/day = 370 mg Cu

•Total = 566 mg Cu/day giving an excess of 430 mg Cu/day

This calculation does not take into account periods of higher feeding levels of pasture, and there may be other factors involved, for example the level of zinc dosing. Data from Gribbles Veterinary Laboratory (Facial Eczema Risk and Incidence Monitor No 2, 18^th^ Jan 2013 – unpublished observations) showed much lower FE spore counts and case incidence compared to the previous season, which could lead to lower levels of zinc being administered and therefore increased copper uptake from the diet. Breed may also play a role in making this herd vulnerable to copper toxicity; some data suggests that Jersey cows accumulated more copper in their livers than Holstein cows fed the same diet [12].

## Conclusions

This case highlighted the key role that liver samples play in diagnosing herd copper status (either as biopsies from live cows or samples taken from cull cows). This herd had experienced deaths from copper toxicity, yet Cu(S) concentrations from healthy animals were within the normal range and did not show any correlation with Cu(L) concentrations (which were above safe levels). However in cows with clinical signs of suspected copper toxicity serum or plasma copper concentration is a cheap and definitive test for copper toxicosis; at this point copper stores are being released from the liver into the bloodstream. This case highlights the small margin of safety above the upper limit of normal for Cu(L), and should be kept in mind when advising on copper supplementation.

Also there is little evidence that there is any production benefit from supplementing additional copper to dairy cattle when liver copper concentration is at the low end of the reference range (95 – 2000 μmol/kg wet tissue [6]). A New Zealand supplementation study [13] showed no difference in milk yield or fertility between dairy cattle with liver copper concentrations of 100 μmol/kg wet tissue compared to 350 μmol/kg wet tissue. An American study [14] also showed no difference in milk yield and consistency between control and supplemented animals; the supplemented animals in this study were split into two groups being supplemented either 10 mg Cu/kg or 40 mg Cu/kg (as CuSO_4_), and at the end of the trial the 40 mg Cu/kg group had liver copper concentrations of more than double the control group. Liver copper concentration was measured in dry tissue rather than fresh tissue so cannot be directly compared with the liver copper concentrations from this case report.

Follow-up liver biopsies following a molybdenum/sulphur drenching program and dietary changes showed a reduction in liver copper reserves, which may support the use of ammonium molybdate and sodium sulphate to reduce storage of copper in the liver. However PKE feeding had stopped four days prior to the first death (at the same time as drying off) and the herd was moved away from the effluent paddock with high pasture copper levels. Therefore due to the lack of a control (undrenched) group it is not possible to know in this cases whether the observed reduction in liver copper concentrations was due to decreasing dietary copper, the use of molybdenum/sulphur drench, or a combination of the two. Ideally supplementation with molybdenum/sulphur would have lasted for 3-4 weeks to produce a greater reduction in Cu(L) [15] but it was not continued due to farmer reluctance (due to his perception of ongoing copper deficiency issues on this farm).

In this case, drying off the herd and the simultaneous associated dietary changes would seem to have been the stress that triggered the copper toxicity outbreak. There were several sources of copper in the diet (PKE, pasture), but the majority of this herd’s daily copper intake came from the mineral blend supplement. All copper supplements to this herd were withdrawn for at least a year and will only be re-introduced in conjunction with close monitoring of herd copper status via liver sampling. With increasing levels of supplement being fed to Irish dairy cows, production animal veterinarians should be aware of the risk of copper toxicity and consider implementing strategies to monitor herd copper status. Also differential diagnoses for cows found dead or moribund should include copper toxicity.

## Abbreviations

Cu(L): Liver copper; Cu(S): Serum copper; DM: Dry matter; FE: Facial eczema; GGT: Gamma-glutamyl transferase; GLDH: Glutamate dehydrogenase; PKE: Palm kernel extract.

## Competing interests

The authors declare that there are no conflicting interests.

## Authors’ contributions

HJ supervised the clinical investigation. LB drafted the paper. NM provided additional advice during the clinical investigation and supervised production of the manuscript. All authors read and approved the final manuscript.

## References

[B1] Parkinson TJ Vermunt JJ Malmo J Diseases of Cattle in Australasia (1st edn) 2010 Wellington, New Zealand: VetLearn®, New Zealand Association Foundation for Continuing Education

[B2] Claypool DW Adams FW Pendell HW Hartmann NA Jr Bone JF Relationship between the level of copper in the blood plasma and liver of cattle J Anim Sci 1975 41 911 914 1158816 10.2527/jas1975.413911x

[B3] Anonymous Quarterly review of diagnostic cases: April to June 2012 Surveillance 2012 39 45 54

[B4] Masters DG Judson GJ White CL Lee J Grace ND Current issues in trace element nutrition of grazing livestock in Australia and New Zealand Aust J Agric Res 1999 50 1341 1364 10.1071/AR99035

[B5] Underwood EJ Suttle NF The Mineral Nutrition of Livestock (3rd edn) 1999 Wallingford, United Kingdom: CABI Publishing

[B6] Grace ND Knowles SO Sykes AR Managing Mineral Deficiencies in Grazing Livestock 2010 Hamilton, New Zealand: Occasional Publication No. 15, New Zealand Society of Animal Production

[B7] Schmidl M Von Forstner D Laboratory Testing in Veterinary Medicine Diagnosis and Clinical Monitoring (3rd edn) 1986 Ingelheim, Germany: Boehringer Mannheim

[B8] Thompson RH Blanchflower WJ Wet-ashing apparatus to prepare biological materials for atomic absorption spectrophotometry Lab Pract 1971 20 859 861 5129403

[B9] Parton K Bruere AN Chambers JP Veterinary Clinical Toxicology (3rd edn) 2006 VetLearn®

[B10] Weaver AD St Jean G Steiner A Bovine Surgery and Lameness (2nd edn) 2005 Hoboken, USA: Blackwell Publishing Ltd

[B11] Alexander P The TG Hungerford Vade Mecum Series for Domestic Animals Series a, No 20 Control and Therapy of Diseases of Cattle 2005 University of Sydney Post-Graduate Foundation in Veterinary Science

[B12] Du Z Hemken RW Harmon RJ Copper metabolism of Holstein and Jersey cows and Heifers fed diets high in cupric sulfate or copper proteinate J Dairy Sci 1996 79 1873 1880 10.3168/jds.S0022-0302(96)76555-4 8923258

[B13] Jolly PD Clark RG Fraser AJ Organising Committee 4th AAAP Congress Effect of Copper Status and Copper Supplementation on Milk Production and Fertility in Dairy Cattle Proceedings of the 4th Asian Australian Animal Production Congress: 1-6 Feb 1987: Hamilton, New Zealand 1987 418

[B14] Engle TE Fellner V Spears JW Copper status, serum cholesterol, and milk fatty acid profile in Holstein cows fed varying concentrations of copper J Dairy Sci 2001 84 2308 2313 10.3168/jds.S0022-0302(01)74678-4 11699463

[B15] Morgan PL Grace ND Lilley DP Using sodium molybdate to treat chronic copper toxicity in dairy cows: a practical approach N Z Vet J 2013 62 167 170 24215634 10.1080/00480169.2013.862150

